# IGF2BP2 Regulates the Progression of Alzheimer's Disease Through m6A‐Mediated NLRP3 Inflammasome

**DOI:** 10.1002/iid3.70121

**Published:** 2025-01-09

**Authors:** Wu Jingrui, Yang Haihui, Yan Jinjin, Fang Le

**Affiliations:** ^1^ College of Medical Technology Xi'an Medical College Xi'an Shaanxi China; ^2^ Depatment of Medical Technology Huyi District Xi'an Shaanxi China; ^3^ Department of Clinical Laboratory Norinco General Hospital Xi'an Shaanxi China

**Keywords:** Alzheimer's disease, Aβ, N6‐methyladenosine, pyroptosis

## Abstract

**Background:**

Recent studies show that N6‐methyladenosine (m6A) plays an important role in the pathogenesis of the Alzheimer's disease (AD), while the mechanisms involved were studied insufficiently.

**Aims:**

The present study aimed to explore the effect of human insulin‐like growth factor 2 (IGF2) mRNA binding proteins 2 (IGF2BP2), one of the m6A‐binding proteins on the progression of AD.

**Materials & Methods:**

The mRNA and protein expression level were determined using RT‐qPCR and western blot, respectively. MTT assay was carried out to evaluate cell viability. The content of ROS, antioxidant enzymes, IL‐1β and pyroptosis, as well as m6A contents were determined using relative commercial kit. The AD models were built using Aβ1‐42 ‐stimulated hippocampal neuron in vitro and AD mice in vivo.

**Results:**

Our results showed that IGF2BP2 was significantly upregulated in the Aβ1‐42 ‐stimulated hippocampal neuron. IGF2BP2 inhibition reversed the decreased cell viability and the increased cell apoptosis induced by Aβ1‐42. IGF2BP2 siRNA transfection alleviated Aβ1‐42 induced pyroptosis and pyroptosis‐related proteins upregulation. we also found that IGF2BP2 inhibition downregulated the expression of NLRP3 through m6A methylation. Furthermore, overexpression of NLRP3 partly reversed the effect of IGF2BP2 inhibition on Aβ1‐42 ‐induced hippocampal neuron injury. In addition, IGF2BP2 improved cognitive function and alleviated Aβ1‐42 neuronal injury in vivo.

**Conclusion:**

Knockdown of IGF2BP2 inhibit neuronal damage and pyroptosis in the hippocampus cells, and improve cognitive function in AD partly through m6A‐mediated NLRP3 inflammasome.

## Graphical abstract

GF2BP2 was increased in Aβ42 induced hippocampal neuron, and the blood and brain of AD mice, knockdown of IGF2BP2 attenuated Aβ‐induced neuron injury through regulating the stability of NLRP3 mRNA.

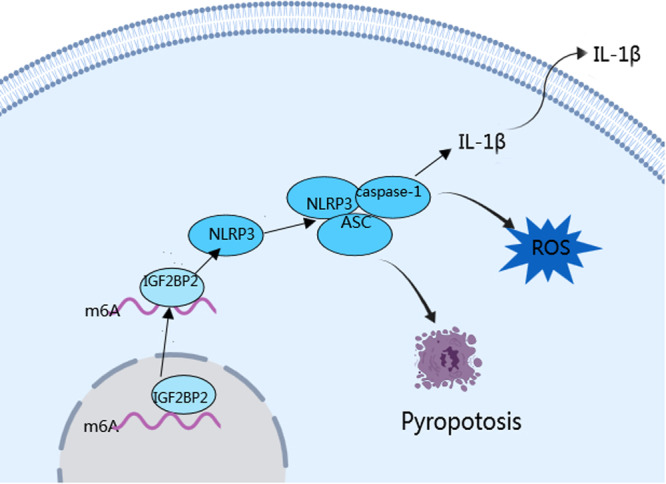

## 1 Introduction

Alzheimer's disease (AD) is a neurodegenerative disease mostly affecting the aged people, especially people over 65 years old [1]. The hallmark pathological feature of AD is neuritic plaques accumulation, neuroinflammation, and cognitive disorder. The synaptic impairment, neuronal dysfunction, and neurofibrillary tangles caused by hyperphosphorylation and cleaved of the microtubule‐associated protein tau, along with the amyloid plaques are two major pathological lesions in the brain [2]. As the main component of amyloid plaques, Amyloid β‐protein (Aβ) plays a crucial role in the development of AD by leading oxidative stress, neuroinflammatory response, and cell apoptosis of neuron. Thus, the strategy aims to prevent Aβ‐induced neuron injury is highly focused.

Pyroptosis is a pro‐inflammatory programmed cell death, which characterized by membrane pore formation, cell swelling and lysis, and cytokine release. There are two pathways that triggers pyroptosis: canonical inflammasome pathway and noncanonical inflammasome pathway. In the canonical inflammasome, inflammasomes is activated and bind to ASC, leading to ASC focus. And then triggers the recruitment of ASC and caspase‐1 to form macromolecular complexes, where caspase‐1 is activated. Activated caspase‐1 contributes to the cleavage of gasdermin D (GSDMD) and liberates N‐terminal fragment of GSDMD (GSDMD‐NT) to form pores in the plasma membrane, leading to the maturation and release of inflammatory mediators. In noncanonical inflammasome pathway, caspase‐4/5/11 is activated by lipopolysaccharide (LPS) derived from bacteria, cleaving GSDMD and inducing pyroptosis by assembling of NOD‐like receptor protein 3 (NLRP3) inflammasome [3]. Pyroptosis and neuroinflammatory response are involved in the pathology of Aβ_1‐_42‐induced neuronal damage and cognitive impairment in AD [4]. The NLRP3 inflammasome was activated in AD, and associated with tau aggregation, Aβ plaques, as well as spatial memory loss, hippocampal synaptic plasticity decrease, other AD‐related symptoms [5].

N6‐methyladenosine (m6A) is a type of internal modification in mRNA, which is involved in the development of the neurodevelopmental disorders. The level of m6A RNA methylation is reported to be high in the mammalian brain, and involved in neurodevelopment and synaptic plasticity [6]. The effect of m6A RNA modification is dynamically and reversibly regulated by related enzymes, including methyltransferases (writers), demethylases (erasers), and m6A binding proteins (readers). Methyltransferase‐like 14 (Mettl14), which is an important methyltransferases of m6A modification, promoted the apoptosis of spinal neurons after spinal cord injury [7], silence of Mettl14 in the substantia nigra region activated microglia and astrocyte, impaired motor function and locomotor activity [8]. Global m6A level is downregulated in PD model both in vivo and in vitro, inhibition of m6A elevate oxidative stress and dopaminergic neuron apoptosis [9, 10]. the m6A methyltransferase methyltransferase‐like 3 (Mettl3), Mettl14, and (Wilms' tumor 1‐associated protein) WTAP were decreased and demethylases AlkB homolog 5 and obesity‐associated protein (FTO) was decreased in the postmortem AD hippocampal tissues [11, 12, 13]. The human insulin‐like growth factor 2 (IGF2) mRNA binding proteins 2 (IGF2BP2), one of the m6A‐binding proteins, plays a role in neuroprotection and tissue remodeling response in the hypoxic‐ischemic‐injured brain [14]. Besides, IGF2BP2 was also reported to be reduced in schizophrenia and bipolar disorder [15]. Recent study indicated that IGF2BP2 was interacted with the novel biomarker of AD. However further research is still required for exploring the effect of IGF2BP2 on the progression of AD.

The present study aimed to explore the role of IGF2BP2 in the development of AD. Both in vitro and in vivo model was built. We first investigated the effect of IGF2BP2 on Aβ1‐42 ‐injured hippocampal neuron and the related mechanism. Then AD mice were used to confirm the results observed in hippocampal neuron.

## 2 Materials and Methods

### 2.1 Primary Hippocampal Neuron Culture and Treatment

Hippocampal neurons were isolated and cultured from C57BL/6 mice brains according to previous study [16]. Briefly, the hippocampal tissue was harvested and dissociated by d‐Hank's solution. After being washed by Dulbecco's modified Eagle's medium (Nutrient Mixture F‐12 Ham [DMEM/F‐12 medium]), the homogenate was mildly centrifuged for 3 min at 2000 rpm (room temperature). The cell pellet was incubated with poly‐d‐lysine, and then the cells was cultured with Neurobasal media supplemented with B27 (Gibco), and 0.5 mM glutamine (Gibco) for 4 h, the medium was replaced by fresh medium.

For Aβ treatment, the isolated hippocampal cells were cultured and exposed to Aβ_1–42_ (GL Biochem, Shanghai, China) for 24 h. And 0.1% DMSO solution was used to treat control group.

### 2.2 Cell Transfection

Three short hairpin RNA (shRNA) against IGF2BP2 (sh‐IGF2BP2‐1, sh‐IGF2BP2‐2, sh‐IGF2BP2‐3, Table S1) and one corresponding scrambled negative control shRNA (sh‐NC) (Genechem, Shanghai, China) were used to silence the expression of IGF2BP2. 2 × 10^6^ hippocampal neurons were cultured each well, and then transfected with 2 µg/mL sh‐IGF2BP2 or shRNA‐NC using Lipofectamine 2000 (Invitrogen, Thermo Fisher Scientific Inc.) according to manufacturer's instructions. The knockdown efficiency was confirmed using western blot assay at 48 h posttransfection, the highest knockdown efficiency cells were used for subsequent experiments.

### 2.3 RT‐qPCR

Cells were harvested and total RNA was extracted using Trizol reagents (Invitrogen, USA). Total of 5 µg RNA was reversely transcribed into cDNA using PrimeScript RT Master Mix (Takara, Japan). Then SYBR Green Master Mix (Takara, Japan) were used to amplify and quantify cDNA using an ABI 7500 instrument (Applied Biosystems) [17]. β‐Actin was used as internal reference. The fold change of gene expression was calculated by using (the $2^{‐∆∆C_{t}}$ method. The primer sequences information is given in Table S2.

### 2.4 Western blot

Total protein was extracted from the cells using RIPA extraction reagent (Beyotime, China) and separated by 12% SDS‐PAGE. Next, the targeted bands were transfected onto polyvinylidene fluoride membrane. After blocking with 5% skim milk, the membrane was incubated with primary antibody and secondary antibody (1:10,000, DY60202; Deeyeebio). The following primary antibodies were used: anti‐IGF2BP2 (1:1000; Cell Signaling Technology), anti‐NLRP3 (1:1000; Abcam), anti‐Caspase‐1 (1:1000; Cell Signaling Technology), anti‐GSDMD‐N (1:1000; Cell Signaling Technology), anti‐ASC (1:1000; Abcam), anti‐β‐actin(1:1000; Abcam). Finally, the membrane was visualized using Enhanced Chemiluminescence (ECL) Kit (Beyotime). The gray values of the protein bands were quantified with the Bio‐Rad image analysis system (Bio‐Rad Laboratories, Hercules, CA, USA).

### 2.5 CCK‐8 Assay

Cells were seeded and cultured in 96‐well plates, CCK‐8 working solution (10 mL) was added to each well till the cells were 80% confluent. After incubating for 3 h, the absorbance value at 450 nm in each well was tested using a microplate reader (Thermo Fisher, USA) [18].

### 2.6 Measurement of ROS

Intracellular ROS levels were measured with a Reactive Oxygen Species Assay Kit (CA1410; Solarbio, Beijing, China) in accordance with the publisher's directions. In brief, DCFH‐DA (10 μmol/L) was added to the cells and incubated for 20 min. The fluorescence intensity was determined using flow cytometry (NovoCyte, Aceabio, USA) and analyzed with FlowJo software.

### 2.7 Detection of Antioxidant Enzymes

The content of malondialdehyde (MDA) and superoxide dismutase (SOD), catalase (CAT), and glutathione (GSH) activity were determined according to the manufacture's instruction of the assay kits (Shanghai Langdun Biotech, Shanghai, China), respectively. Cells were harvested and then treated according to the relative manufacturer's instruction. The OD values were measured at 450 nm.

### 2.8 Pyroptosis Analysis

The apoptosis was determined using the FAM‐FLICA in vitro Caspase‐1 Detection Kit (ImmunoChemistry, Bloomington, MN, USA). In brief, cells were collected and stain with propidium iodide and FAM‐FLICA, and then tested using CytoFLEX flow cytometry, and data analysis was performed with the software.

### 2.9 Enzyme‐Linked Immunosorbent Assay (ELISA)

The level of IL‐1β was detected using Mouse IL‐1 beta/IL‐1F2 Quantikine ELISA Kit (R&D Systems Inc.) following the instruction of the manufacturer. Briefly, the cells were collected and treated with trypsin, and then added with the reagent provided by the ELISA kits. The absorbance was read at 450 nm wavelength.

### 2.10 m6A Content Measurement

The total m6A content was determined using an m6A methylation assay kit (Abcam, ab185912) [19]. The extracted RNA was incubated with the binding solution, capture antibody, and detection antibody, the absorbance was determined after being cultured with enhancer solution at 450 nm wavelength.

### 2.11 RNA Immunoprecipitation (RIP) Assays

RIP assay was performed using a Magna RIP RNA Binding Protein Immnoprecipitation kit (Millipore) according to the publisher's directions. hippocampal neurons were lysed using RIP lysis buffer and then incubated with antibodies against IGF2BP2 (Abcam), or IgG (Abcam). The RNAs bound to immunoprecipitated IGF2BP2 were extracted and detected by qRT‐PCR.

### 2.12 Animal

APP695/PS1‐dE9 (APP/PS1) double transgenic mice and wild‐type (WT) littermates (the Model Animal Research Center of Nanjing University, Nanjing, China) at 2‐month‐old (25–30 g) were used in the study. The mice were randomly divided into three groups: WT (n = 8), APP/PS1, APP/PS1 + vehicle (n = 8), and APP/PS1 + aav‐shIGF2BP2 (n = 8). Mice were injected via the tail vein with 1 × 10^9^ PFU aav‐shIGF2BP2. All animal experiments complied with ARRIVE guidelines, and were carried out in accordance with the National Institute of Health Guide for the Care and Use of Laboratory Animals. This study was formally approved by the Animal Care and Use Committee of the 521 Hospital of Ordnance Industry.

### 2.13 Morris Water Maze (MWM) Test

MWM test was performed to evaluate the cognitive behavior of mice. To test spatial reference memory, the pool was filled with tap water mixed with nontoxic white paint, and divided into four quadrants, the mice were trained for 5 days with four trials per day per mouse to find the platform in water for 60 s. If the mouse failed to find the submerged platform on a given trial, the mouse was guided to the platform and kept there for 10 s. After 5 days of training, the platform was removed, and mice were allowed to swim freely in the pool for 60 s. The escape latency, time spent in quadrant, crossing frequency, and distance of target quadrant were recorded.

### 2.14 Nissl Staining

The whole brain was obtained after behavior assays. The sections were fixed in a 4% paraformaldehyde PBS solution and then made as paraffin sections using a microtome. The sections were stained with Nissl staining (Beyotime Institute of Biotechnology, China) [20]. Finally, the fixed samples were visualized under a microscope (optical; Leica Microsystems, Wezlar, Switzerland).

### 2.15 Immunofluorescence Staining

The Immunofluorescence was performed on cryostat sections. After being repaired the antigen and permeabilizing with Triton, the sections were then blocked with 5% normal serum for 2 h and incubated at 4°C for 36 h with anti‐NeuN (Abcam, 1:1000, Rabbit). The nuclear DNA was Labelled using DAPI. Fluorescent signals analysis was performed using a fluorescence microscope (Olympus).

### 2.16 Statistical Analysis

All experiments were repeated three times, and the data was analyzed by SPSS 20.0 statistical software. Data are expressed as means ± standard deviation (SD). The correlation was analyzed by Pearson's correlation test. Statistical significance was assessed using Student's *t*‐test and one‐way ANOVA, and *p* < 0.05 was considered to be statistically significantly relevant.

## 3 Results

### 3.1 Knockdown of IGF2BP2 Inhibited Neuron Injury in the Aβ1‐42‐Stimulated Hippocampal Neuron

**Figure 1:**
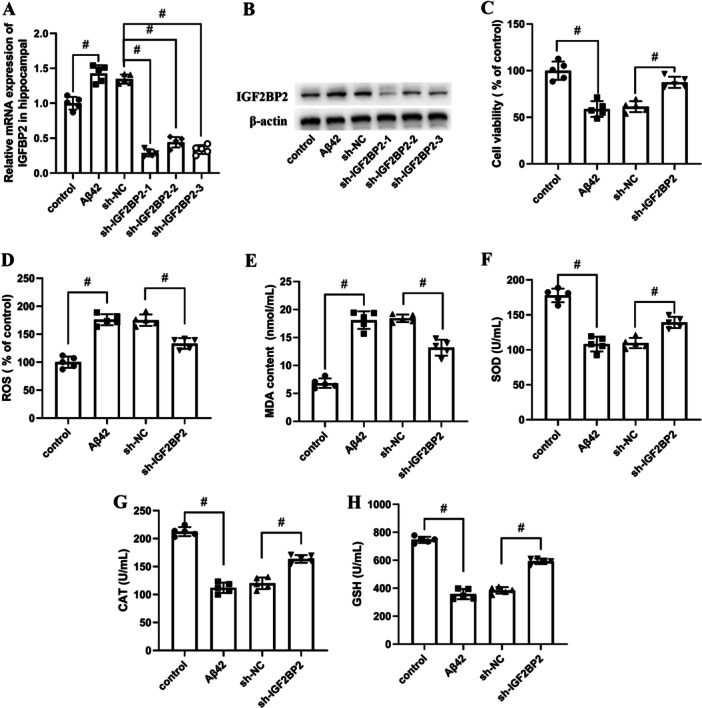
Effect of IGF2BP2 inhibition on neuron injury in the Aβ1‐42‐stimulated hippocampal neuron. Cells were treated with Aβ1‐42 for 24 h, (A) The mRNA expression and (B) protein expression level of IGF2BP2 was detected using RT‐qPCR and western blot. (C) CCK8 was used to identify the cell viability. (D) Intracellular ROS level was determined by DCFH‐DA assay and analyzed by fluorescence microscopy. The activity of (E) MDA, (F) SOD, (G) CAT, and (H) GSH were assessed with the relative ELISA kit. All data in this figure represent the means ± SEM of three independent experiments. Results are expressed as mean ± SD, Between‐group data were analyzed using one‐way ANOVA, *n* = 5. ^#^ *p* < 0.05.

As shown in Figure 1A, IGF2BP2 was found to be significantly upregulated in the Aβ1‐4 stimulated hippocampal neuron. To figure out the effect of IGF2BP2 on Aβ1‐42 ‐stimulated hippocampal neuron, IGF2BP2 siRNAs were used to silence the expression of IGF2BP2. The results indicated that sh‐IGF2BP2‐1, sh‐IGF2BP2‐2, and sh‐IGF2BP2‐3 all significantly suppressed the expression of IGFBL2, in which sh‐IGF2BP2‐1 exhibited the best efficiency. Thus sh‐IGF2BP2‐1 was used in the subsequently experiments (Figure 1A,B). IGF2BP2 inhibition dramatically reversed the decreased cell viability induced by Aβ1‐42 (Figure 1C). Besides, IGF2BP2 inhibition suppressed the levels of ROS and MDA in the Aβ1‐42 ‐stimulated hippocampal neuron (Figure 1D,E). Meanwhile, the levels of SOD, CAT and GSH which were decreased by Aβ1‐42 were significantly elevated by IGF2BP2 siRNA (Figure 1F–H).

### 3.2 IGF2BP2 Depletion Blocked Pyroptosis Induced by Aβ

**Figure 2:**
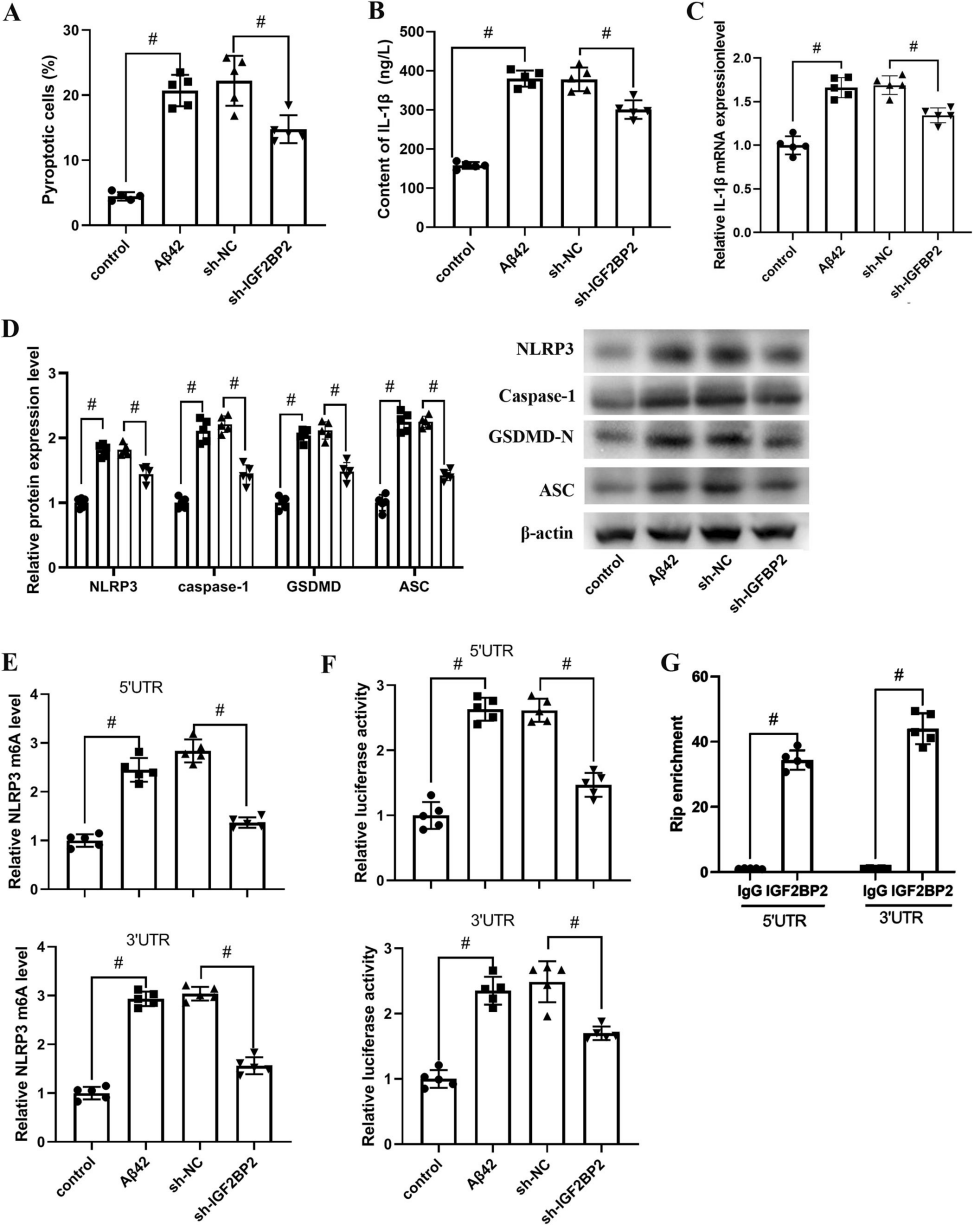
Effect of IGF2BP2 inhibition on pyroptosis in the Aβ1‐42 ‐stimulated hippocampal neuron. Cells pre‐transfected with sh‐NC or sh‐IGF2BP2 were treated with Aβ1‐42 for 24 h. (A) The pyroptotic cells were analyzed using Caspase‐1 Detection Kit. (B) The content of IL‐1β was measured using Elisa kits. (C) The mRNA expression of IL‐1βwas tested by RT‐qPCR assay. (D) The protein expression of NLRP3, caspase‐1, GSDMD, and ASC were determined by western blot. (E) The m6A level of NLRP3 5′‐UTR and 3′‐UTR was examined by MeRIP‐qPCR analysis. (F) The luciferase activity was determined. (G) RIP and qRT‐PCR were used to test the binding of IGF2BP2 and NLRP3. Results are expressed as mean ± SD, Between‐group data were analyzed using Student's *t*‐test and one‐way ANOVA, *n* = 5. ^#^ *p* < 0.05.

Next, the role of IGF2BP2 in neuron pyroptosis was explored. the results revealed that Aβ1‐42 remarkable promoted pyroptosis, which was markedly inhibited by sh‐IGF2BP2 (Figure 2A). The content and mRNA level of IL‐1β was significantly increased by Aβ treatment, which were decreased by in IGF2BP2 siRNA in hippocampal neuron (Figure 2B,C). Furthermore, Aβ1‐42 treatment remarkedly upregulated the pyroptosis‐related proteins, including NLRP3, caspase‐1, GSDMD, and ASC, while IGF2BP2 siRNA transfection significantly alleviated the upregulation of the pyroptosis‐related proteins that induced by Aβ1‐42 (Figure 2D). As IGF2BP2 is a m6A reader, we speculated that there might be relationship between IGF2BP2‐regulated m6A modification and NLRP3. The data showed that IGF2BP2 siRNA transfection decreased both 3′ and 5′ UTR of NLRP3 m6A modification which increased by Aβ1‐42 (Figure 2E). Moreover, the results of dual‐luciferase assays implicated that the levels of 5′‐UTR and 3′‐UTR of NLRP3 were increased by Aβ1‐42, IGF2BP2 siRNA inhibited the levels of 5′‐UTR and 3′‐UTR of NLRP3 in Aβ1‐42 treated neuron (Figure 2F). RIP enrichment analysis showed that RIP enrichment was significantly higher in anti‐IGF2BP2 antibody group than anti‐IgG antibody, indicating that IGF2BP2 direct bind to NLRP3 (Figure 2G).

### 3.3 IGF2BP2 Silence Inhibits Neuron Injury and Cell Pyroptosis by Targeting Nlrp3

**Figure 3:**
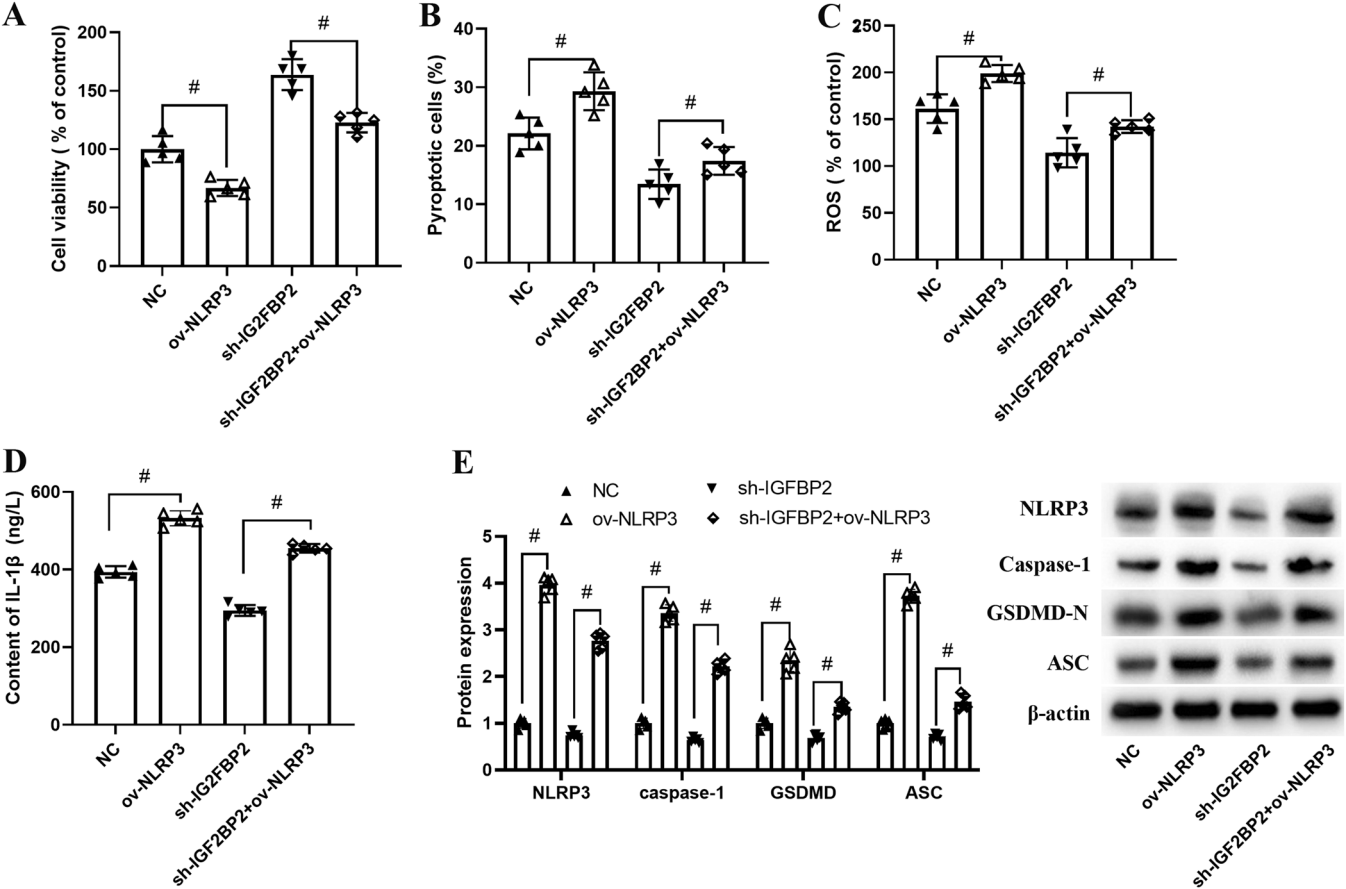
The involvement of NLRP3 in IGF2BP2 silence regulated neuron injury and cell pyroptosis. Cells cotransfected with sh‐IGF2BP2 or ov‐NLRP3 were treated with Aβ1‐42 for 24 h. (A) Cell viability was assessed by CCK8 assay. (B) The pyroptotic cells were analyzed using Caspase‐1 Detection Kit. (C) Intracellular ROS level was determined by DCFH‐DA assay and analyzed by fluorescence microscopy. (D) The content of IL‐1β was measured using Elisa kits. (E) The protein expression of NLRP3, caspase‐1, GSDMD, and ASC were determined by western blot. Results are expressed as mean ± SD, between‐group data were analyzed using one‐way ANOVA, *n* = 5. ^#^ *p* < 0.05.

To confirm the role of NLRP3 in IGF2BP2‐regulated neuron injury and cell pyroptosis, gain function of NLRP3 was experimented. As shown in Figure 3A,B, IGF2BP2 inhibition promoted the cell viability and decreased pyroptotic cells, while overexpression of NLRP3 inhibited cell viability, contributed to cell pyroptosis, and attenuated IGF2BP2 inhibition induced cell viability elevation and cell pyroptosis. The content of ROS and IL‐1β in ov‐NLRP3 group was remarkable higher than NC group, and higher in sh‐IGF2BP2+ov‐NLRP3 group than sh‐IGF2BP2 group (Figure 3C,D). Besides, ov‐NLRP3 upregulated the protein expression of pyroptosis‐related protein, and partly reversed the downregulated protein expression of pyroptosis‐related protein that induced by IGF2BP2 siRNA (Figure 3E).

### 3.4 Knockdown of IGF2BP2 Improved Cognitive Function in AD Mice

**Figure 4:**
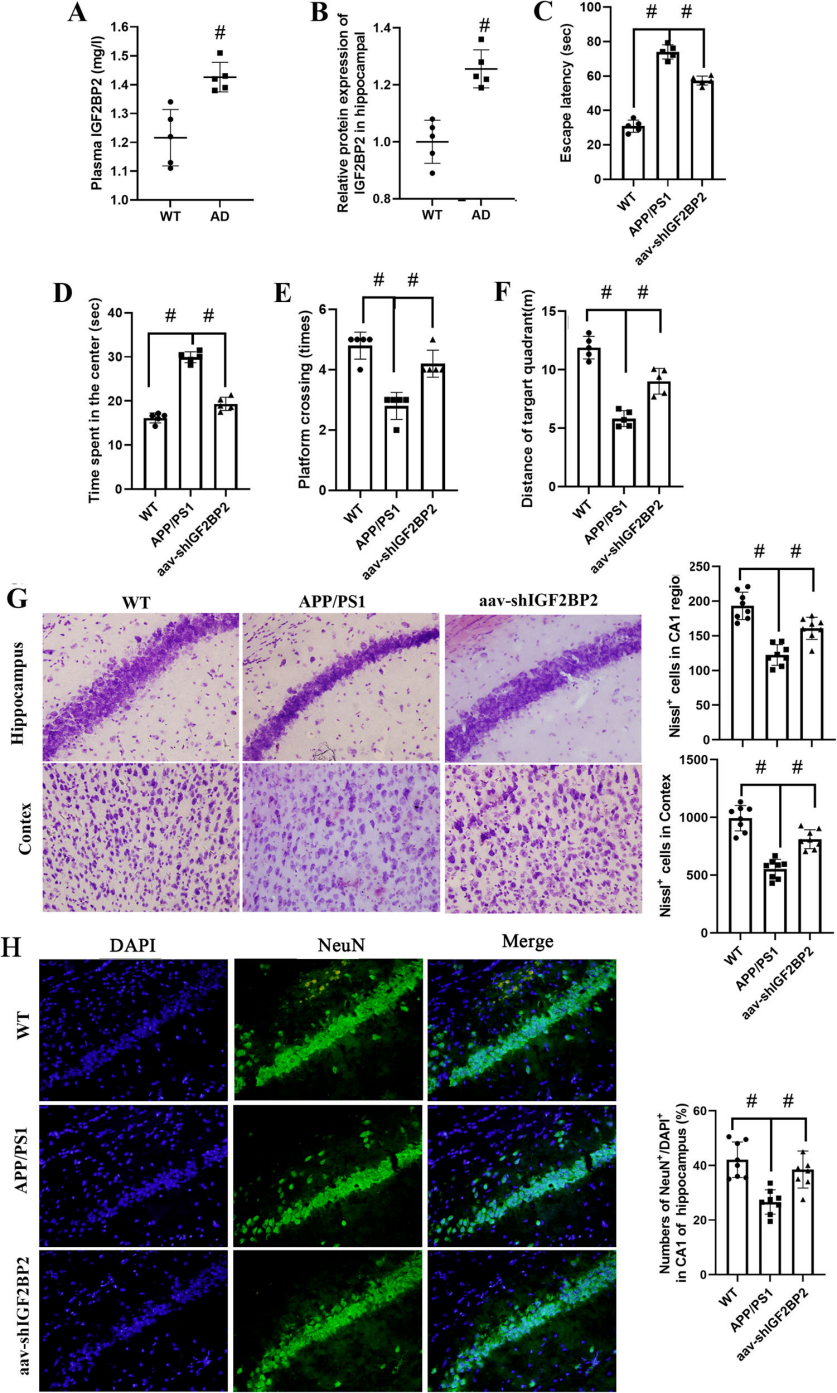
Effect of IGF2BP2 inhibition on neuron injury and cognitive function in AD mice. aav‐shIGF2BP2 was intracranially injected into mice via the tail vein the hippocampus. (A) The mRNA and (B) protein expression level of IGF2BP2 was detected using RT‐qPCR and western blot. (D) The escape latency time, (E) the spent in the center, (F) frequency of crossing, and (G) distance in target quadrant were evaluated using Morris water maze test. (G) The results of Nissl staining in the cortex and hippocampus (400). (H) The results of the representative immunofluorescence images of NeuN (green) and DAPI (blue) from CA1 of hippocampus, and the bars showed the counted number of NeuN‐positive cells. Results are expressed as mean ± SD, Between‐group data were analyzed using Student's *t*‐test (A, B) and one‐way ANOVA (C–F), *n* = 5. ^#^ *p* < 0.05.

The role of IGF2BP2 in the progression of AD was further examined in vivo using APP/PS1 mice. we first detected the expression level of IGF2BP2 in the blood and hippocampal of AD mice. The results implied that IGF2BP2 was significantly high expressed both in the blood and hippocampal of AD mice (Figure 4A,B). Then aav‐shIGF2BP2 was injected to explore the effect of IGF2BP2 on the cognitive dysfunction of AD mice. We see that the escape latency and the time spent in the center was markedly longer in APP/PS1 mice compare with aav‐shIGF2BP2 group and WT mice (Figure 4C,D). Also, aav‐shIGF2BP2 elevated the platform crossing times and the distance in target quadrant in APP/PS1 mice (Figure 4E,F). The results of nissl staining indicated that aav‐shIGF2BP2 significant increased neuronal counts (Figure 4G). Besides, compared to the APP/PS1 group, the level of NeuN positive cells in CA1 region of mice brain were significantly elevated by aav‐shIGF2BP2 (Figure 4H).

## 4 Discussion

The m6A modification is very rich in the brain, and it is reported that dysregulation of m6A is involved in brain development and the neural degenerative diseases [21]. As one of the readers of m6A modification, IGF2BP2 was upregulated in the substantia nigra of the PD mice [22]. Our study found that the expression of IGF2BP2 was elevated in Aβ1‐42 induced hippocampal neuron, and the blood and brain of AD mice. The similar results were also reported by Deng et al. [23] who indicated that IGF2BP2 was high expressed in the brain tissues of Alzheimer's patients. Furthermore, the expression of IGF2BP2 was upregulated in the entorhinal cortex, hippocampus, postcentral gyrus, and superior frontal gyrus of AD patients. Thus, there might be a role of IGF2BP2 in the pathogenesis and development of AD.

IGF2BP2 is implicated in the nervous system disease. Repression of IGF2BP2 inhibited cell viability, cell proliferation, metastases, drug‐resistance, and tumor‐forming capacity in glioblastoma [24, 25, 26, 27]. And the expression of IGF2BP2 is positively related with the poor prognosis of glioblastoma [28]. A study focused on hypoxic‐ischemic brain injury revealed that IGF2BP2 could interacted by RBM3 and thus enhances neuronal differentiation potential and limited neuronal apoptosis [29]. Inhibition of IGF2BP2 promoted astrocytic differentiation and suppressed the neurogenic potential of early‐stage neural precursor cells, while IGF2BP2 promoted the neurogenic potential and suppressed astrocytic differentiation of late‐stage neural precursor cells, while there was no different on cell proliferation of IGF2BP2 on neural precursor cells [30]. IGF2BP2 inhibits axon regeneration, which are immunoreactive to tau [31]. Similarly, our study found that IGF2BP2 silence inhibited Aβ_1‐42_‐induced hippocampal neuron injury and cell pyroptosis. These indicated that there might be a role of IGFBP2 in the pathogenesis of AD.

Recently study reported that pyroptosis is increased in the cerebrospinal fluid of patients with AD and induced by Aβ1‐_42_ through NLRP3/caspase‐1/GSDMD pathway [32, 33]. The accumulation of Aβ induce activation of inflammasome and trigger caspase‐1‐dependent neuronal pyroptosis in brain [34]. Meanwhile, neuroinflammation attenuation of AD in the deactivation pathway of pyroptosis have been observed and thus improved cognitive impairment [32]. Our study suggested that IGF2BP2 inhibition attenuated neuronal pyroptosis induced by Aβ. These results were similar to the study of Wang et al., who found that silence of IGF2BP2 inhibited the activity of GADMD and caspase‐1 in LPS‐injured Beas‐2B cell [35].

m6A modification change the expression of genes that associated with the development of neuron system diseases, including AD, PD, depression, cerebral apoplexy, epilepsy [21]. IGF2BP2 is involved in multiple biological processes through regulating the localization, stability, and translation of RNAs [36]. Results of the present study indicated that IGF2BP2 inhibition attenuated the m6A methylation of NLRP3. As the same, IGF2BP1 was indicated to negative regulated the stability of NLRP3 through binding to the 5′‐UTR and 3′‐UTR of the NLRP3 mRNA [37]. Another study demonstrated that the stability of NLRP3 was increased by METTL14 through promoting the m6A methylation of NLRP3 mRNA in an IGF2BP2‐dependent manner, and there is the direct binding of IGF2BP2 to NLRP3 mRNA [38]. Token together, IGF2BP2 regulated the neuronal pyroptosis by mediating NLRP3 RNA stability through m6A methylation. Ge et al. [39] reported that IGF2BP2 could interact with NLRP3 mRNA and inhibit the m6A level of NLRP3 in microglia, and thus to promote the neuroinflammation in ischemic stroke. Inconsistent with that, the present study focused on the neuronal pyroptosis in AD, and conclude that IGF2BP2 could regulate the neuronal pyroptosis by mediating NLRP3 RNA stability through m6A methylation. Similarly, the study of Deng et al. [23] indicated that IGF2BP2 may increase PKP2 through m6A association mechanism, and thus lead to the occurrence of AD. All in all, IGF2BP2 may involve in the occurrence and development of AD partly via modifying the m6A methylation of the related genes.

In conclusion, our current study first explored the role of IGF2BP2 in mediating neuronal pyroptosis and cognitive impairment in vitro. The results provide evidence that IGF2BP2 was increased in Aβ1‐42 induced hippocampal neuron, and the blood and brain of AD mice, knockdown of IGF2BP2 may attenuated Aβ‐induced neuron injury partly through regulating the stability of NLRP3 mRNA.

However, we have not tested the specific effect of IGF2BP2 on the accumulation of Aβ and phosphorylation of tau in AD mice, nor have we explored the complex regulatory network of IGFBP2 in modulating the development of AD. Further studies are needed to confirm the effect and enrich the regulatory mechanism of IGF2BP2 in the development of AD.

## Author Contributions

**Wu Jingrui:** carried out the experiments and wrote the manuscript. **Yang Haihui:** carried out most of the experiments. **Yan Jinjin:** acquisition, analysis, and interpretation of data for the work. **Le Fang:** conception and design of the work, revised the manuscript.

## Ethics Statement

All animal experiments complied with ARRIVE guidelines, and were carried out in accordance with the National Institute of Health Guide for the Care and Use of Laboratory Animals. This study was formally approved by the Animal Care and Use Committee of the 521 Hospital of Ordnance Industry.

## Conflicts of Interest

The authors declare no conflicts of interest.

## Supporting information

Supporting information.

Supporting information.

## Data Availability

All data generated or analyzed during this study are included in this published article.

## References

[1] K. A. Matthews , W. Xu , A. H. Gaglioti , et al., “Racial and Ethnic Estimates of Alzheimer's Disease and Related Dementias in the United States (2015–2060) in Adults Aged >/=65 Years,” Alzheimer's & Dementia 15, no. 1 (2019): 17–24. 10.1016/j.jalz.2018.06.3063 PMC6333531 30243772

[2] N. N. Naseri , H. Wang , J. Guo , M. Sharma , and W. Luo , “The Complexity of Tau in Alzheimer's Disease,” Neuroscience Letters 705 (2019): 183–194. 31028844 10.1016/j.neulet.2019.04.022 PMC7060758

[3] T. Du , J. Gao , P. Li , et al., “Pyroptosis, Metabolism, and Tumor Immune Microenvironment,” Clinical and Translational Medicine 11, no. 8 (2021): e492. 34459122 10.1002/ctm2.492 PMC8329701

[4] Y. Huang , X. Li , G. Luo , et al., “Pyroptosis as a Candidate Therapeutic Target for Alzheimer's Disease,” Frontiers in Aging Neuroscience 14 (2022): 996646. 36185484 10.3389/fnagi.2022.996646 PMC9520296

[5] J. Barczuk , N. Siwecka , W. Lusa , W. Rozpędek‐Kamińska , E. Kucharska , and I. Majsterek , “Targeting NLRP3‐Mediated Neuroinflammation in Alzheimer's Disease Treatment,” International Journal of Molecular Sciences 23, no. 16 (2022): 8979. 36012243 10.3390/ijms23168979 PMC9409081

[6] J. Widagdo and V. Anggono , “The m6A‐Epitranscriptomic Signature in Neurobiology: From Neurodevelopment to Brain Plasticity,” Journal of Neurochemistry 147, no. 2 (2018): 137–152. 29873074 10.1111/jnc.14481

[7] G. Gao , Y. Duan , F. Chang , T. Zhang , X. Huang , and C. Yu , “METTL14 Promotes Apoptosis of Spinal Cord Neurons by Inducing EEF1A2 m6A Methylation in Spinal Cord Injury,” Cell Death Discovery 8, no. 1 (2022): 15. 35013140 10.1038/s41420-021-00808-2 PMC8748977

[8] Y. Teng , Z. Liu , X. Chen , et al., “Conditional Deficiency of m6A Methyltransferase Mettl14 in Substantia Nigra Alters Dopaminergic Neuron Function,” Journal of Cellular and Molecular Medicine 25, no. 17 (2021): 8567–8572. 34288397 10.1111/jcmm.16740 PMC8419180

[9] X. Chen , C. Yu , M. Guo , et al., “Down‐Regulation of m6A mRNA Methylation Is Involved in Dopaminergic Neuronal Death,” ACS Chemical Neuroscience 10, no. 5 (2019): 2355–2363. 30835997 10.1021/acschemneuro.8b00657

[10] M. Han , Z. Liu , Y. Xu , et al., “Abnormality of m6A mRNA Methylation Is Involved in Alzheimer's Disease,” Frontiers in Neuroscience 14 (2020): 98. 32184705 10.3389/fnins.2020.00098 PMC7058666

[11] F. Zhao , Y. Xu , S. Gao , et al., “METTL3‐Dependent RNA m(6)A Dysregulation Contributes to Neurodegeneration in Alzheimer's Disease through Aberrant Cell Cycle Events,” Molecular Neurodegeneration 16, no. 1 (2021): 70. 34593014 10.1186/s13024-021-00484-x PMC8482683

[12] H. Huang , J. Camats‐Perna , R. Medeiros , V. Anggono , and J. Widagdo , “Altered Expression of the m6A Methyltransferase METTL3 in Alzheimer's Disease,” eNeuro 7, no. 5 (2020): ENEURO.0125‐20.2020. 10.1523/ENEURO.0125-20.2020 PMC7540926 32847866

[13] Z. Lv , T. Xu , R. Li , et al., “Downregulation of m6A Methyltransferase in the Hippocampus of Tyrobp (‐/‐) Mice and Implications for Learning and Memory Deficits,” Frontiers in Neuroscience 16 (2022): 739201. 35386591 10.3389/fnins.2022.739201 PMC8978996

[14] L. Fletcher , E. Isgor , S. Sprague , et al., “Spatial Distribution of Insulin‐Like Growth Factor Binding Protein‐2 Following Hypoxic‐Ischemic Injury,” BMC Neuroscience 14 (2013): 158. 24359611 10.1186/1471-2202-14-158 PMC3911968

[15] C. Weissleder , M. J. Webster , G. Barry , and C. Shannon Weickert , “Reduced Insulin‐Like Growth Factor Family Member Expression Predicts Neurogenesis Marker Expression in the Subependymal Zone in Schizophrenia and Bipolar Disorder,” Schizophrenia Bulletin 47, no. 4 (2021): 1168–1178. 33274367 10.1093/schbul/sbaa159 PMC8266571

[16] M. Hamanoue , Y. Ikeda , T. Ogata , and K. Takamatsu , “Predominant Expression of N‐Acetylglucosaminyltransferase V (GnT‐V) in Neural Stem/Progenitor Cells,” Stem Cell Research 14, no. 1 (2015): 68–78. 25524127 10.1016/j.scr.2014.11.004

[17] X. L. Shi , N. Yan , Y. J. Cui , and Z. P. Liu , “A Unique GSK‐3β Inhibitor B10 Has a Direct Effect on Aβ, Targets Tau and Metal Dyshomeostasis, and Promotes Neuronal Neurite Outgrowth,” Cells 9, no. 3 (2020): 649. 32155989 10.3390/cells9030649 PMC7140427

[18] M. Qu , L. Zuo , M. Zhang , et al., “High Glucose Induces Tau Hyperphosphorylation in Hippocampal Neurons via Inhibition of ALKBH5‐mediated Dgkh m(6)A Demethylation: A Potential Mechanism for Diabetic Cognitive Dysfunction,” Cell Death & Disease 14, no. 6 (2023): 385. 37385994 10.1038/s41419-023-05909-7 PMC10310746

[19] Y. Dagan , Y. Yesharim , A. R. Bonneau , et al., “m6A Is Required for Resolving Progenitor Identity During Planarian Stem Cell Differentiation,” EMBO Journal 41, no. 21 (2022): e109895. 35971838 10.15252/embj.2021109895 PMC9627665

[20] J. Kou , M. Wang , J. Shi , et al., “Curcumin Reduces Cognitive Deficits by Inhibiting Neuroinflammation Through the Endoplasmic Reticulum Stress Pathway in Apolipoprotein E4 Transgenic Mice,” ACS Omega 6, no. 10 (2021): 6654–6662. 33748578 10.1021/acsomega.0c04810 PMC7970496

[21] J. Lv , L. Xing , X. Zhong , K. Li , M. Liu , and K. Du , “Role of N6‐methyladenosine Modification in Central Nervous System Diseases and Related Therapeutic Agents,” Biomedicine & Pharmacotherapy 162 (2023): 114583. 36989722 10.1016/j.biopha.2023.114583

[22] Z. Yu , L. Huang , Y. Xia , et al., “Analysis of m6A Modification Regulators in the Substantia Nigra and Striatum of Mptp‐Induced Parkinson's Disease Mice,” Neuroscience Letters 791 (2022): 136907. 36209975 10.1016/j.neulet.2022.136907

[23] Y. Deng , H. Zhu , L. Xiao , C. Liu , Y. L. Liu , and W. Gao , “Identification of the Function and Mechanism of m6A Reader IGF2BP2 in Alzheimer's Disease,” Aging 13, no. 21 (2021): 24086–24100. 34705667 10.18632/aging.203652 PMC8610118

[24] Q. Mu , L. Wang , F. Yu , et al., “Imp2 Regulates GBM Progression by Activating IGF2/PI3K/Akt Pathway,” Cancer Biology & Therapy 16, no. 4 (2015): 623–633. 25719943 10.1080/15384047.2015.1019185 PMC4622833

[25] M. S. Lindstrom , “Expanding the Scope of Candidate Prognostic Marker IGFBP2 in Glioblastoma,” Bioscience Reports 39, no. 7 (2019): BSR20190770. 31296788 10.1042/BSR20190770 PMC6639463

[26] K. W. Lin , A. Liao , and A. A. Qutub , “Simulation Predicts IGFBP2‐HIF1α Interaction Drives Glioblastoma Growth,” PLoS Computational Biology 11, no. 4 (2015): e1004169. 25884993 10.1371/journal.pcbi.1004169 PMC4401766

[27] Y. Liu , C. Song , F. Shen , J. Zhang , and S. W. Song , “IGFBP2 Promotes Immunosuppression Associated With Its Mesenchymal Induction and FcgammaRIIB phosphorylation in Glioblastoma,” PLoS One 14, no. 9 (2019): e0222999. 31560714 10.1371/journal.pone.0222999 PMC6764691

[28] M. Janiszewska , M. L. Suvà , N. Riggi , et al., “Imp2 Controls Oxidative Phosphorylation and Is Crucial for Preserving Glioblastoma Cancer Stem Cells,” Genes & Development 26, no. 17 (2012): 1926–1944. 22899010 10.1101/gad.188292.112 PMC3435496

[29] X. Zhu , J. Yan , C. Bregere , et al., “RBM3 Promotes Neurogenesis in a Niche‐Dependent Manner via IMP2‐IGF2 Signaling Pathway after Hypoxic‐Ischemic Brain Injury,” Nature Communications 10, no. 1 (2019): 3983. 10.1038/s41467-019-11870-x PMC6726629 31484925

[30] Y. Fujii , Y. Kishi , and Y. Gotoh , “IMP2 Regulates Differentiation Potentials of Mouse Neocortical Neural Precursor Cells,” Genes to Cells 18, no. 2 (2013): 79–89. 23331702 10.1111/gtc.12024

[31] S. Blizard , D. Park , N. O'Toole , et al., “Neuron‐Specific IMP2 Overexpression by Synapsin Promoter‐Driven AAV9: A Tool to Study Its Role in Axon Regeneration,” Cells 10, no. 10 (2021): 2654. 34685634 10.3390/cells10102654 PMC8534607

[32] C. Han , Y. Yang , Q. Guan , et al., “New Mechanism of Nerve Injury in Alzheimer's Disease: β‐Amyloid‐Induced Neuronal Pyroptosis,” Journal of Cellular and Molecular Medicine 24, no. 14 (2020): 8078–8090. 32521573 10.1111/jcmm.15439 PMC7348172

[33] H. Shen , C. Han , Y. Yang , et al., “Pyroptosis Executive Protein GSDMD as a Biomarker for Diagnosis and Identification of Alzheimer's Disease,” Brain and Behavior 11, no. 4 (2021): e02063. 33587329 10.1002/brb3.2063 PMC8035446

[34] M. S. Tan , L. Tan , T. Jiang , et al., “Amyloid‐β Induces NLRP1‐Dependent Neuronal Pyroptosis in Models of Alzheimer's Disease,” Cell Death & Disease 5, no. 8 (2014): e1382. 25144717 10.1038/cddis.2014.348 PMC4454321

[35] J. Wang , X. Yuan , and N. Ding , “IGF2BP2 Knockdown Inhibits LPS‐Induced Pyroptosis in BEAS‐2B Cells by Targeting Caspase 4, a Crucial Molecule of the Non‐Canonical Pyroptosis Pathway,” Experimental and Therapeutic Medicine 21, no. 6 (2021): 593. 33884031 10.3892/etm.2021.10025 PMC8056110

[36] N. Dai , J. Rapley , M. Angel , M. F. Yanik , M. D. Blower , and J. Avruch , “mTOR phosphorylates IMP2 to Promote IGF2 mRNA Translation by Internal Ribosomal Entry,” Genes & Development 25, no. 11 (2011): 1159–1172. 21576258 10.1101/gad.2042311 PMC3110954

[37] J. Lan , B. Xu , X. Shi , Q. Pan , and Q. Tao , “WTAP‐Mediated N(6)‐Methyladenosine Modification of NLRP3 mRNA in Kidney Injury of Diabetic Nephropathy,” Cellular & Molecular Biology Letters 27, no. 1 (2022): 51. 35761192 10.1186/s11658-022-00350-8 PMC9235192

[38] X. Yuan , T. Li , L. Shi , J. Miao , Y. Guo , and Y. Chen , “Human Umbilical Cord Mesenchymal Stem Cells Deliver Exogenous miR‐26a‐5p via Exosomes to Inhibit Nucleus Pulposus Cell Pyroptosis Through METTL14/NLRP3,” Molecular Medicine 27, no. 1 (2021): 91. 34412584 10.1186/s10020-021-00355-7 PMC8375162

[39] P. Ge , H. Duan , C. Tao , et al., “TMAO Promotes NLRP3 Inflammasome Activation of Microglia Aggravating Neurological Injury in Ischemic Stroke Through FTO/IGF2BP2,” Journal of Inflammation Research 16 (2023): 3699–3714. 37663757 10.2147/JIR.S399480 PMC10473438

